# Supplementary material for: The genome sequence of the Dark Moss-moth, Bryotropha affinis (Haworth, 1828) (Lepidoptera: Gelechiidae)
Source: Wellcome Open Res. 2026 Oct 2;11:789. [Version 1] doi: 10.12688/wellcomeopenres.28017.1 (PMC13684025; doi:10.12688/wellcomeopenres.28017.1)
Supplement: Peer review history

# Reviewer report: Reviewer response for version 1

**DOI:** 10.21956/wellcomeopenres.30822.r173167

**Published:** 2026-10-26

**Authors:** Jurate De Prins

**Affiliations:** 1 Belgian Institute of Natural Sciences, Brussels, Belgium; 2 Flemish Entomological Society, Vrieselhof, Belgium

I reviewed many articles for Wellcome Sanger, and I have certain considerations that are applicable not only to this article but in general.

**Verification of the sample which is studied**

 In this case, at least the specimen is photographed from a dorsal view, but since it is in poor shape, there are significant doubts that it is *Bryotropha affinis*. 10 species of Bryotropha occur just across the channel in Flanders, of which two are not photographed yet. Looking at the subapical stripes on the forewings in the presented Fig. 1, they more closely resemble a species from Tortricidae: Olethreutinae than a gelechiid. No doubt that whole genomes are needed, but the problem is that if they are based on misidentified specimens, then all future genomic studies will be calibrated on an initial misidentification. To avoid this, the specimens that are studied should be set so that all the characters are well seen and compared. The specimen was collected in London, so why not set it properly and comfortably at the NHM, to avoid any doubt about its ID?

**Meiotic transformations**

 It is very good that meiotic transformation and meiotic trivalent, with two Z chromosomes, Z1 and Z2, are presented; n=32 is an unusual chromosome number in a meiotic set of microlepidoptera. At this stage, the world has now switched to whole-mitogenome sequencing and therefore more explanation is needed about the possible scenario of sex trivalent formation and its impact on speciation.

**COPE principles** https://publicationethics.org/

 The world has also moved to adopt and promote integrity in scholarly publications. Young and not-so-young authors are sensitive and aware that their work is credited, known, and acknowledged. Leading authorship is important in terms of copyrights and responsibility. I reviewed many articles from Wellcome Sanger. No matter who the leading author is, they are written according to the same template, same wording, same presentation. Standardisation is welcome, but research articles are more than methodological reports. My plea is to adopt the COPE https://publicationethics.org/ policy.

Are the datasets clearly presented in a usable and accessible format, and the assembly and annotation available in an appropriate subject-specific repository?

Yes

Are sufficient details of the sequencing and extraction, software used, and materials provided to allow replication by others?

Partly

Are the rationale for sequencing the genome and the species significance clearly described?

Yes

Are the protocols appropriate and is the work technically sound?

Partly

Reviewer Expertise:

Taxonomy, Karyology, Lepidoptera, Bioinformatics

I confirm that I have read this submission and believe that I have an appropriate level of expertise to confirm that it is of an acceptable scientific standard, however I have significant reservations, as outlined above.

# Reviewer report: Reviewer response for version 1

**DOI:** 10.21956/wellcomeopenres.30822.r174614

**Published:** 2026-10-20

**Authors:** Liang Lü

**Affiliations:** 1 Hebei Normal University, Shijiazhuang, China

The manuscript presents a genome assembly from an individual female *Bryotropha affinis. *The workflow of Darwin Tree of Life Barcoding is very mature and changeless, so I do not have any comments on that. The authors identified 32  chromosomes based on HiC phasing results, but I suggest **c**hromosome karyotype analysis that may calibrate this result.

Are the datasets clearly presented in a usable and accessible format, and the assembly and annotation available in an appropriate subject-specific repository?

Yes

Are sufficient details of the sequencing and extraction, software used, and materials provided to allow replication by others?

Yes

Are the rationale for sequencing the genome and the species significance clearly described?

Yes

Are the protocols appropriate and is the work technically sound?

Yes

Reviewer Expertise:

phylogenomics and entomology

I confirm that I have read this submission and believe that I have an appropriate level of expertise to confirm that it is of an acceptable scientific standard.

# Reviewer report: Reviewer response for version 1

**Published:** 2026-10-02

**Authors:** Automated Benchmarking

**Affiliations:** 1 Wellcome Sanger Institute, Hinxton, UK

As this article is a Genome Note, Automated Benchmarking has been used to determine whether the reported assembly passes four defined checks established by the Earth BioGenome Project: the raw and assembled data are publicly available, the Earth BioGenome Project three-part metric is met, the k-mer completeness exceeds 90%, and the BUSCO score passes the stated threshold. For Bryotropha affinis (ilBryAffi1), all four checks passed. Data available: pass. ENA study PRJEB89605, raw-read accessions ERR14995226 and ERR15164872, and assembly accessions GCA_965641975.1 and GCA_965641925.1 are public. Earth BioGenome Project three-part metric: pass. Haplotype 1 is reported as 6.C.Q61, meeting the 6.C.Q40 reference standard. k-mer completeness: pass. The combined haplotype assemblies have 99.61% k-mer completeness, exceeding the Earth BioGenome Project target of 90%. BUSCO: pass. Haplotype 1, which is proposed as the reference assembly for this species, has 97.9% complete BUSCOs (95.6% single-copy and 2.4% duplicated). BUSCO software version: 5.8.3; lineage dataset: lepidoptera_odb10. It meets the target of greater than 90% complete and single-copy BUSCOs. This decision uses values reported in the Genome Note and public accession records.

Reviewer Expertise:

NA

I confirm that I have read this submission and believe that I have an appropriate level of expertise to confirm that it is of an acceptable scientific standard.
